# A Famous Chinese Medicine Formula: Yinhuo Decoction Antagonizes the Damage of Corticosterone to PC12 Cells and Improves Depression by Regulating the SIRT1/PGC-1*α* Pathway

**DOI:** 10.1155/2022/3714857

**Published:** 2022-03-04

**Authors:** Hongdan Xu, Shurong Xing, Xia Lei, Jinyue Yi, Shuang Liu, Yanqiu Du, Bo Yang, Ning Zhang

**Affiliations:** ^1^Department of Pharmacy, Wuxi Higher Health Vocational Technology School, Wuxi 214000, China; ^2^Chinese Medicine & Postdoctoral Mobile Research Station, Heilongjiang University of Chinese Medicine, Harbin 150040, China; ^3^Rehabilitation Medical College, Jiamusi University, Jiamusi 154000, China; ^4^Institute of Traditional Chinese Medicine, Wuxi Traditional Chinese Medicine Hospital, Wuxi 214000, China; ^5^College of Pharmacy, Heilongjiang University of Chinese Medicine, Harbin 150040, China

## Abstract

The study aimed to explore the antidepressant effect of Yinhuo Decoction and further to explore its underlying molecular mechanism acting on depressant. Here, high-performance liquid chromatography (HPLC) analysis was used to the composition analysis. Postmenopausal depression (PMD) model and corticosterone (CORT)-induced cell model were constructed. Adrenal coefficient and hematoxylin and eosin staining were applied to assess changes in the adrenal glands. MTT staining, Hoechst 33342 staining, and JC-1 fluorescence staining were used to detect the PC12 activity and apoptosis. CORT and oxidative stress indicators were measured using commercial kits. Western blot and immunohistochemical were used to detect the protein expression of GCR. In addition, genes related to SIRT1/PGC-1*α* pathway were also tested. In PMD model mice, Yinhuo Decoction evidently increased adrenal coefficient and relieved adrenal lesions. Meanwhile, we observed that Yinhuo Decoction reduced the CORT and GCR levels. In CORT-treated PC12 cells, Yinhuo Decoction remarkably reduced cytotoxicity and apoptosis. Besides, Yinhuo Decoction attenuated the oxidative stress response. Mechanically, we confirmed that Yinhuo Decoction reduced CORT-induced PC12 damage by regulating SIRT1/PGC-1*α* pathway. Thus, we concluded that Yinhuo Decoction antagonized CORT-induced injury in PC12 cells and improved depression in PMD mice. This provided a new direction for the treatment of depression.

## 1. Introduction

Depression is a chronic and life-threatening mental illness caused by a variety of factors. Its main symptom is a psychological or emotional disorder, affecting approximately 350 million people around the world [1, 2]. Notably, suffering from depression also increases the risk of potentially fatal diseases, such as cardiovascular and cerebrovascular diseases and neurodegenerative diseases [3]. According to reports, major depression is ranked as the third most common disease burden in the world and is predicted to rank first by 2030 [4, 5]. Although a large number of antidepressants have been developed for clinical use, their therapeutic efficacy is far from satisfactory. Further, only about 30% of patients with major depression respond to current antidepressant, while about 70% of responders do not achieve the complete remission [6] Therefore, more and more researchers began to pay attention to the pathogenesis of depression and drugs with potential therapeutic targets.

Considering the complex pathogenesis of diseases and the characteristics of traditional Chinese medicines (TCM) with multiple components and targets, more and more Western researchers are accepting TCM formulas that have been used in China for thousands of years [7]. The compatibility of TCM can increase potency, reduce toxicity, or produce new pharmacological effects that are not present in any single herb medicine [8]. Yinhuo Decoction is a famous Chinese medicine formulas developed by Chen Shiduo, a physician in the Qing Dynasty. The formula consists of five kinds of TCM, namely, Rehmannia glutinosa, Morinda officinalis, Ophiopogon japonicus, Poria, and Schisandra [9]. A previous clinical study has depicted that Yinhuo Decoction can treat a variety of brain diseases, such as insomnia. After taking Yinhuo Decoction, patients fall asleep better and sleep longer [10]. In addition, after treatment with Yinhuo Decoction for autistic patients, the communication barriers and behavior prevention of the patients improved compared with before, and the symptoms of autism were also alleviated [11]. However, the antidepressant effect of Yinhuo Decoction was rarely reported, and its mechanism is still unclear, which limits in-depth and comprehensive understanding of it.

Depression has always been considered as a disease related to stress and long-term exposure to stress will cause a variety of depressive symptoms. Moreover, patients with depression had different degrees of dysfunction of the hypothalamic-pituitary-adrenal (HPA) axis [12]. These evidences indicated that the HPA axis dysfunction was the main pathological feature of mood disorders and also the target of drug intervention. Here, the study aimed to explore the antidepressant effect of Yinhuo Decoction and further to explore its underlying molecular mechanism acting on depressant through stress injury-related depression models in vivo and in vitro. This research will provide theoretical support and scientific basis for the Yinhuo Decoction in the treatment of depression.

## 2. Materials and Methods

### 2.1. Preparation of Yinhuo Decoction

The raw herbs were purchased from Beijing Tongrentang Harbin Drugstore and authenticated by Prof. Xiaozhong Chen from Heilongjiang University of Chinese Medicine, China. The herbs were tested to be qualified according to the requirements of Chinese Pharmacopeia (2015 Edition). Through single factor investigation and orthogonal experiment, we optimized the factors such as soaking time, decocting time, adding water amount, and obtained the best preparation method of Yinhuo Decoction. Briefly, Rehmannia glutinosa, Morinda officinalis, Ophiopogon japonicus, Poria, and Schisandra were mixed at the ratio of 3 : 2 : 2 : 10 : 1 : 1 (15 g:10 g:10 g:5 g:5 g). Then, 8 times of the compound dose of water was added to soak the raw herbs for 2 h, and water decocted and extracted twice for 1 h. The mixed extract was filtered and concentrated at 50°C lyophilized to yield Yinhuo Decoction powder and then diluted in DMEM medium.

### 2.2. High-Performance Liquid Chromatography (HPLC) Analysis

The analysis of the constituents was performed on an Agilent HPLC (1260 infinity) equipped with a diode array detector (1260 variable wavelength detector) with an XBridgeVR C18 column (250 × 4.6 mm, 5 *μ*m). The chromatographic separations were carried out on a mobile phase consisting of 0.1% acetic acid (a) and acetonitrile (b) with a linear gradient as follows: 0–30 min, 10%–20% B; 30–50 min, 20%–40% B; 50–51 min, 40%–10% B; 51–60 min, 10% B. The flow rate was 1 mL/min, the detection wavelength was 254 nm, and the inject volume was 10 *μ*L.

The Yinhuo Decoction extract (0.1 g) was placed in a volumetric flask with 10.0 mL of 95% methanol, during which the mixture was subjected to ultrasonication for 15 min twice. Authentic samples were prepared in methanol solution (1.0 mg/mL). All the samples were filtered through a 0.22 *μ*m filter (Millipore) before they were injected into the HPLC. Peaks were confirmed using the UV absorption and retention times of the authentic samples.

### 2.3. Animals and Groups

Totally, 30 ICR mice (weight, 25 ± 5 g) provided by GLP Experimental Center of Heilongjiang University of Traditional Chinese Medicine were included in the present study [SCXK (Hei) 2013-0004]. All animals were free to access the water and kept in a clean animal room with temperature and humidity of 22–24°C and 50–60%. The study was approved by the ethics committee of Heilongjiang University of Chinese Medicine. All procedures were in strict accordance with the recommendations in the Guide for the Care and Use of Laboratory Animals of the National Institutes of Health.

The mice were randomly divided into 6 groups: Sham group, Model group, Fluoxetine group, Yinhuo Decoction group, SIRT1 inhibitor group, and PGC-1*α* inhibitor group. In the Sham group, the same size cellulite was taken back into the ovaries after ovaries were exposed, and distilled water was given by gavage. In the Model group, mice underwent ovariectomy (OVX) surgery followed by intragastric administration of distilled water. In the Fluoxetine group and Yinhuo Decoction group, the mice were also treated with OVX surgery, followed by intragastric administration of 2.34 g•mL^−1^ Yinhuo Decoction and Fluoxetine, respectively. In the SIRT1 inhibitor group, the Model mice were treated with Yinhuo Decoction+SIRT1 inhibitor. In the PGC-1*α* inhibitor group, the model mice were treated with Yinhuo Decoction+PGC-1*α* inhibitor.

### 2.4. Cell Culture

Rat pheochromocytoma cells (PC12) were obtained from American Type Culture Collection (ATCC, Manassas, VA). After resuscitation, they were cultured in RPMI-1640 medium containing 10% fetal bovine serum (FBS, Beyotime, China) at 37°C in a humidified atmosphere of 5% CO_2_. When cells reached 70%–80% confluences, they were harvest for subsequent analysis.

### 2.5. Postmenopausal Depression (PMD) Model

Bilateral OVX combined with chronic unpredictable mild stimulation (CUMS) were used to construct PMD model [13]. For OVX, after anesthesia, the mice were fixed on a hard plate with supine position. Then, an incision was made in the abdomen, and the ovaries on both sides were tied with medical sutures and then removed. The method of determining successful ovariectomy is the vaginal epithelium keratosis test: 5 days continuous monitoring the rat vagina did not find the estrous cycle. For CUMS, all mice were housed in a single cage after the surgery, and after 7 days' recovery from surgery, they were treated with CUMS s for 21 days. The following seven stimuli were employed: fasting for 24 h, water deprivation for 24 h, damp pad stimulation for 24 h, tilting cage (45° angle tilt), shaking cage (30 min/cage/time), black and white inversion (7 : 00–19 : 00 black, 19 : 00–7 : 00 white), and ultrasonic stimulation (20 min) for 3 consecutive weeks.

### 2.6. Adrenal Coefficient

After adrenal glands were removed from mice of indicated groups, the wet weight was weighed and calculated according to the following formula: adrenal coefficient = adrenal gland weight (mg)/body weight (g) × 100%.

### 2.7. Hematoxylin and Eosin (H&E) Staining

After OVX, the vaginal secretions of each group of mice were directly dehydrated, transparent, and smeared on a slide. The adrenal gland was fixed in 4% paraformaldehyde for 24 h. Subsequently, the sample tissues were embedded in paraffin and cut into 5 *μ*m sections with a microtome. Dewaxing and rehydration were performed with a xylene and ethanol aqueous solution, followed by H&E staining by conventional method [14]. Sections were stained with hematoxylin for 5 min and eosin for 3 min at 37°C. Finally, the s tissues were observed under a light microscope.

### 2.8. Cell Viability Analysis

MTT assay was conducted to detect cell viability [15]. Briefly, the logarithmic PC12 cells were digested, counted, and diluted to 1 × 10^5^ cells/mL. Then the cells were inoculated to 96-well plates at 180 *μ*L per well. To screen the treatment conditions for CORT-induced PC12 cell damage, PC12 cells were treated with different concentrations of CORT (50 *μ*mol/L, 100 *μ*mol/L, 200 *μ*mol/L, 400 *μ*mol/L, and 800 *μ*mol/L) for different times (6 h, 12 h, 24 h, and 48 h), and then were incubated with MTT at room temperature. To screen the effective protective concentration of Yinhuo Decoction on CORT damaged PC12 cells, they were treated with different concentrations of Yinhuo Decoction (1 mg/mL, 2.5 mg/mL, 5 mg/mL, 7.5 mg/mL, and 10 mg/mL) for 24 h, and then were incubated with MTT at room temperature. After incubation for 4 h, an appropriate amount of DMSO (Hebei Bio-high Technology Deve Co., LID, China) was added and oscillated on the oscillator for 15 min. The optical density at 490 nm was measured with a multifunctional microplate reader (Synergy Neo, BioTek, USA), and data were expressed as absorbance.

### 2.9. Hoechst 33342 Staining

First, the treated PC12 cells were cultured in 6-well plates. After washing twice with PBS, 500 *μ*L Hoechst 33342 dye solution (Beyotime Biotechnology, China) was added to each well and incubated for 15 min at room temperature in the dark. Subsequently, the cells were observed for staining under a fluorescence microscope.

### 2.10. JC-1 Fluorescence Measurement of the Mitochondrial Membrane Potential (MMP)

The MMP of PC12 was examined using JC-1 detection Kits (KeyGEN Bio TECH Co., Ltd.) according previous study [16]. First, the 1 × 105 test PC12 cells were resuspended and incubated with 500 *μ*L JC-1 reagent solution at 37°C in the dark for 15 min. Then, the cells were washed twice with JC-1 buffer. After rinsing, another 1 mL JC-1 buffer was added to resuscitate the cells, and then fluorescence spectrophotometer was used to detect the cells.

### 2.11. Measurement of CORT, Lactate Dehydrogenase (LDH), Superoxide Dismutase (SOD), Glutathione Peroxides (GPx), and Adenosine 5′-Triphosphate (ATP)

The concentration of CORT, LDH, SOD, GPx, and ATP in the PC12 cells were assessed using commercially available ELISA kit (USCN Business Co., Ltd, Wuhan, China), Cytoxicity Detection Kit (Roche, Basel, Switzerland), SOD kit (Nanjing Jiancheng Bioengineering Institute), GPx assay kit, and ATP Assay Kit, respectively, according to the manufacturer's instructions.

### 2.12. Quantitative Real-Time PCR

According to the previous description [17], total RNA was extracted from samples using TRIzol^®^ reagent (Invitrogen; Thermo Fisher Scientific, Inc.). Total RNA was reverse transcribed into cDNA using the PrimeScript™ RT Reagent Kit (Takara Bio, Inc.). Reverse transcription was performed at 16°C for 30 min, 42°C for 30 min, and 85°C for 5 min. Subsequently, SYBR^®^ Premix Ex Taq™ (Takara Biotechnology Co., Ltd.) was used to examine the gene expression. The qPCR was performed using the following thermocycling conditions:

94°C for 6 min, 35 cycles of 96°C for 20 sec, 58°C for 40 sec, and extension at 72°C for 2 min. The relative gene expression was determined using the 2^−*ΔΔ*Cq^ method [18]. The primer sequences for this experiment were as follows: SIRTI sense, GCTTCTTGGAGACTGCGATG and antisense, TGGCAACTCTGATAAATGAAC; PGC-1*α* sense, GACCGTCCAAAGCATTCA and antisense, GACTCATCCTTAGCCTCC; BNDF sense, TGACAAGGCGAAGGGTTTCT and antisense, CGTGCTCAAAAGTGTCAGCC; GADPH sense, CTACCTCATGAAGATCCTGACC and antisense, CACAGCTTCTCTTTGATGTCAC.

### 2.13. Western Blot

According to the manufacturer's protocol [19], proteins were extracted using RIPA Lysis Buffer (Beyotime, China) and protein concentration was measured using a bicinchoninic acid assay (Beyotime, China). Subsequently, the prepared protein was separated by 12% polyacrylamide-SDS gels and then transferred onto PVDF membranes (Roche, Switzerland). After blocked with skim milk, the PVDF membranes were subjected to incubation with primary antibodies: Bcl-2, Bax, cleaved-Caspased-3, GCR, SIRT1, PGC-1*α*, and BDNF (1 : 500, Wanleibio Wuhan, China) overnight at 4°C. On the following day, the membranes were incubated with the secondary antibody at 37°C for 45 min and the intensity of protein expression was detected by ECL chemiluminescence (Beyotime, Beijing, China). Protein expression levels were semiquantified using ImageJ software (version 1.8.0; National Institutes of Health).

### 2.14. Immunohistochemistry (IHC) Staining

According to the previous description [20], the brain tissues were formalin-fixed and paraffin-embedded, sliced into 4 *μ*m-thick sections. Graded ethanol was used to rehydrate the sections after deparaffinization in xylene at room temperature. After washing with PBS, the sections were placed in 3% hydrogen peroxide for 20 min to inhibit endogenous peroxidase, followed by antigen retrieval by heating for 30 min in a microwave. After being blocked with 10% goat sera, the tissue sections were incubated with primary anti-GCR antibody (1 : 500, Cell Signaling Technology, MA, USA) and at 4°C overnight. On the following day, the tissues were washed with PBS and incubated with secondary antibody anti-Rabbit lgG (MaiXin Bio, China). In the negative controls, the primary antibody was replaced by PBS. Finally, the samples were counterstained with DAB (KT1009a, Abgent) and sealed with glass slide by resin. Image Pro Plus 6.0 was used to measure the immunohistochemical staining positive area and cumulative optical density (IOD), and IOD (SUM)/Area (SUM) was used as the final measurement value.

### 2.15. Statistical Analysis

All the data were analyzed by Statistical Package for Social Sciences 20.0 (SPSS, Chicago, IL, USA) and presented as mean ± standard deviation. One-way ANOVA followed by Dunnett's multiple comparison was applied to assess the differences between the groups. *P* < 0.05 indicated significant difference between groups.

## 3. Results

### 3.1. Chemical Characterization Analysis

**Figure 1:**
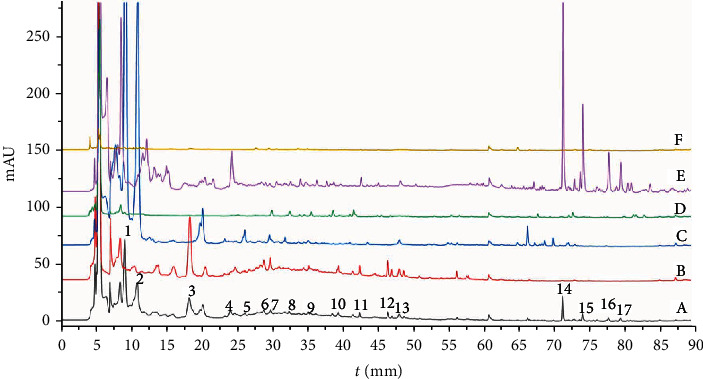
Chemical characterization analysis. (a) Yinhuo Decoction; (b) Rehmannia glutinosa; (c) Morinda officinalis; (d) Ophiopogon japonicus; (e) Schisandra; and (f) Poria. Peaks were assigned based on the UV absorption and retention times of the authentic samples.

The composition in the standard decoction of Yinhuo Decoction was assessed using the HPLC to provide chemical information regarding this formula. In addition, we established chemical fingerprinting and compared the chemical fingerprintings of different batches of Yinhuo Decoction for quality control. As shown in Figure 1, in the sample solution, peaks 1, 2, and 5 were characteristic peaks of Morinda officinalis; peaks 3, 6, 10, and 12 were characteristic peaks of Rehmannia glutinosa; peaks 4, 14, 15, 16, and 17 were characteristic peaks of Schisandra; peak 7 was the common peak of Rehmannia glutinosa, Morinda officinalis, and Ophiopogon japonica; peak 8 was the production peak of Morinda officinalis and Schisandra; peak 9 was the common peak of Ophiopogon japonicus and Schisandra; peak 11 was the common peak of Rehmannia glutinosa and Schisandra; peak 13 was the common peak of Rehmannia glutinosa, Morinda officinalis, and Schisandra.

### 3.2. Yinhuo Decoction Increased Adrenal Coefficient and Relieved Adrenal Lesions in PMD Mice

**Figure 2:**
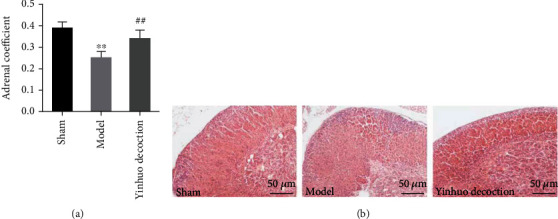
Yinhuo Decoction increased adrenal coefficient and relieved adrenal lesions in PMD model mice. (a) The adrenal coefficient in indicated groups and adrenal coefficient = adrenal gland weight (mg)/body weight (g) × 100%. (b) H&E staining was used to evaluate the pathological changes of adrenal glands in each group. Magnification = 200x, scale = 50 *μ*m; PMD: postmenopausal depression; H&E: hematoxylin and eosin staining; ^∗∗^*P* < 0.01 indicated vs. Sham group; ^##^*P* < 0.01 indicated vs. Model group.

As depicted in Figure 2(a), the adrenal coefficient of the Model group was significantly lower than that of the Sham group. However, after treatment with Yinhuo Decoction, the adrenal coefficient of mice increased significantly as comparison to the Model group. Meanwhile, we examined the structural state of the adrenal glands. Specifically, in the Sham group, the adrenal serosa was intact, the structures of cortical globular zona, zona fasciculata and zona reticulata were clear, the zona fasciculata cells were arranged in bundles, the staining was uniform, and the nucleoli was centered. However, the adrenal cortex area of Model mice was thickened, the zona fasciculata area was thickened, and the cells arrangement were disordered, the cytoplasm was lightly stained, and the nucleus was shifted and pyknotic as comparison to Sham group. Of note, the morphology of adrenal gland of mice in Yinhuo Decoction group was normal, which was similar to that in the Sham group (Figure 2(b)).

### 3.3. Yinhuo Decoction Reduced the Levels of CORT, Total GCR in the Brain, GCR in the Hippocampus DG, and GCR in the Hypothalamus

**Figure 3:**
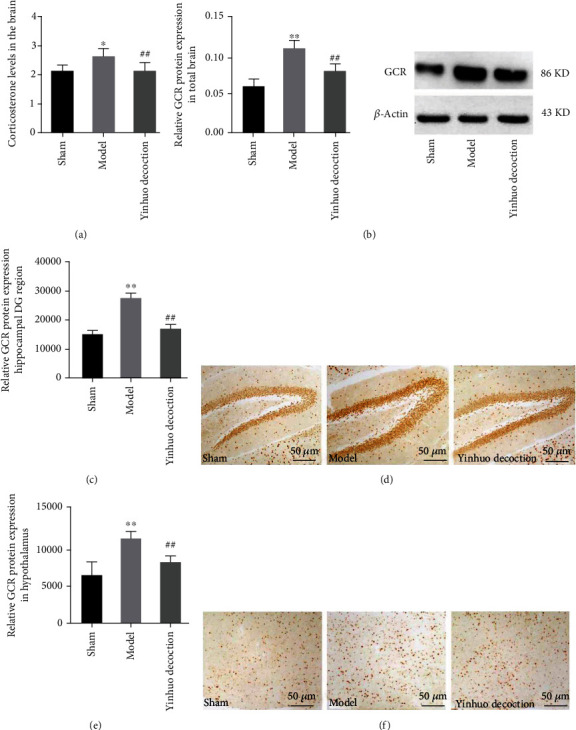
Yinhuo Decoction reduced the levels of CORT, total GCR in the brain, GCR in the hippocampus DG, and GCR in the hypothalamus. (a) Total CORT level in the brain was detected via using ELISA. (b) The GCR protein level in the whole brain was detected by western blot. (c and d) The GCR protein level in hippocampus DG was detected by IHC. (e–f) GCR protein level in hypothalamus was detected by IHC. Magnification = 200x, scale = 50 *μ*m; CORT: corticosterone; GCR: glucocorticoid receptor; ^∗^*P* < 0.05 or ^∗∗^*P* < 0.01 indicated vs. Sham group; ^##^*P* < 0.01 indicated vs. Model group.

In Figure 3(a), we clearly observed that the level of CORT was evidently higher in the Model group than in the Sham group. Interestingly, when the Yinhuo Decoction was added, the content of intracellular CORT was evidently reduced. In addition, the changes in total GCR in the brain, hippocampus DG, and hypothalamus of PMD model mice were also detected. As represented in Figures 3(b)–3(f), both protein data and immunohistochemical data showed that the expression of GCR was remarkably increased in the Model group, while Yinhuo Decoction inhibited the expression of GCR by opposite effect.

### 3.4. Yinhuo Decoction Reduced the Damage of PC12 Cells Induced by CORT

**Figure 4:**
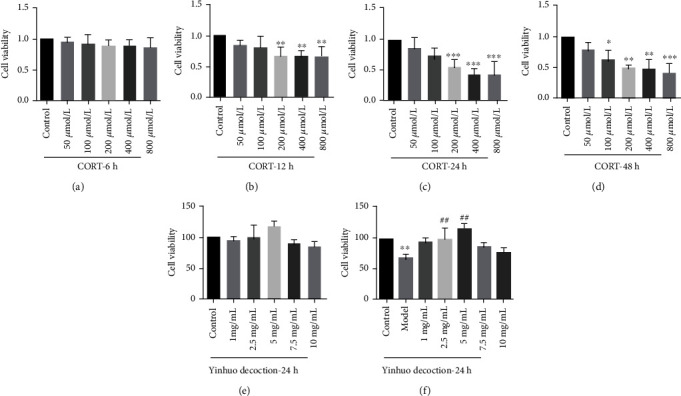
Yinhuo Decoction reduced the damage of PC12 cells induced by CORT. (a) After treatment with different concentration of CORT for 6 h, MTT was conducted to detect PC12 cells viability. (b) After treatment with different concentration of CORT for 12 h, MTT was conducted to detect PC12 cells viability. (c) After treatment with different concentration of CORT for 24 h, MTT was conducted to detect PC12 cells viability. (d) After treatment with different concentration of CORT for 48 h, MTT was conducted to detect PC12 cells viability. (e) Cell activity was measured at 24 h after the PC12 were treated with different concentrations of Yinhuo Decoction. (f) After corresponding treatment, PC12 cell activity was measured by MTT. ^∗^*P* < 0.05, or ^∗∗^*P* < 0.01, or ^∗∗∗^*P* < 0.01 indicated vs. Control group; ^##^*P* < 0.01 indicated vs. Model group.

To screen out the appropriate treatment conditions for CORT to damage PC12 cells, the cells were treated with different concentration gradients of CORT for 6 h, 12 h, 24 h, and 48 h. MTT results showed that cell viability decreased with increasing exposure time and concentration of CORT. When the concentration of CORT was 200 *μ*mol/L and the treatment time was 24 h, the growth status of PC12 cells was poor, and the cell viability decreased to about 50%. Therefore, this condition was selected as the treatment condition of CORT-damaged PC12 cells (Figures 4(a)–4(d)). To screen out the appropriate protective concentration of Yinhuo Decoction, PC12 cells were treated with Yinhuo Decoction of different concentration gradients for 24 h to observe whether the Yinhuo Decoction could damage PC12 cells, and the data revealed that Yinhuo Decoction was nontoxic to PC12 cells and had the best growth condition when the concentration was 5 mg/mL (Figure 4(e)). Then, we treated PC12 cells that had been treated with 200 *μ*mol/L CORT with different concentrations of Yinhuo Decoction for 24 h. Intuitively, when the concentration of Yinhuo Decoction was 5 mg/mL, the cell viability of PC12 cells treated with CORT was significantly increased (Figure 4(f)). Therefore, this condition was selected as the treatment condition of Yinhuo Decoction to protect PC12 cells.

### 3.5. Yinhuo Decoction Reduced the Apoptosis of PC12 Cells Treated with CORT

**Figure 5:**
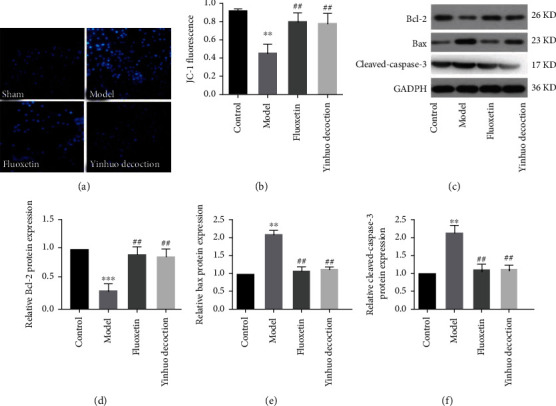
Yinhuo Decoction reduced the apoptosis of PC12 cells treated with CORT. (a) Hoechst 33342 staining was applied to detect the PC12 apoptosis. (b) JC-1 fluorescence was used to determine the MMP in indicated groups. (c) Apoptosis-related proteins expression (Bcl-2, Bax, and Cleaved-Caspase-3) was detected via western blot. (d) Quantitative detection of Bcl-2 protein expression in indicated groups. (e) Quantitative detection of Bax protein expression in indicated groups. (f) Quantitative detection of Cleaved-Caspase-3 protein expression in indicated groups; MMP, mitochondrial membrane potential; ^∗∗^*P* < 0.01 or ^∗∗∗^*P* < 0.001 indicated vs. Control group; ^##^*P* < 0.01 indicated vs. Model group.

Next, we examined the effect of Yinhuo Decoction on PC12 cell apoptosis. As shown in Figure 5(a), the nuclei of neurons in the Control group showed a normal blue color and the nuclei were full, while the neuron nuclei in the Model group showed bright blue, pyknosis, and dense staining. Interestingly, when the PC12 cells were treated with Yinhuo Decoction, the nucleus of cells in the Yinhuo Decoction group returned to normal blue. Meanwhile, JC-1 fluorescence data revealed that the mitochondrial membrane potential of the Model group decreased significantly, whereas the Yinhuo Decoction sharply increased the intracellular mitochondrial membrane potential (Figure 5(b)). Further, we detected the level of apoptosis-related proteins. As expected, Yinhuo Decoction significantly reduced transcription and translation levels of Bax and Cleaved-Caspase-3. Conversely, Yinhuo Decoction remarkably increased the transcription and translation levels of Bcl-2 (Figures 5(c)–5(f)).

### 3.6. Yinhuo Decoction Attenuated the Oxidative Stress of PC12 Cells Treated with CORT

**Figure 6:**
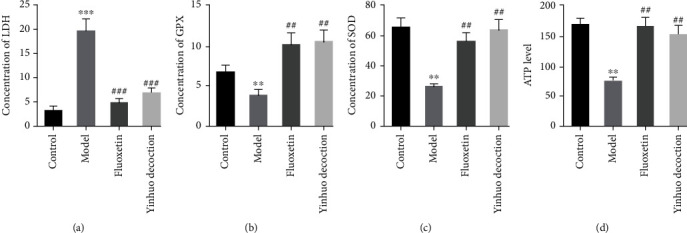
Yinhuo Decoction attenuated the oxidative stress response of PC12 cells treated with CORT. (a) The level of LDH was detected via Cytoxicity Detection Kit. (b) The level of GPx was detected via GPx assay kit. (c) The level of SOD was detected via SOD kit. (d) The level of ATP was detected via ATP Assay Kit. LDH: lactate dehydrogenase; SOD: superoxide dismutase; GPx: glutathione peroxides; ATP: adenosine 5′-triphosphate; ^∗∗^*P* < 0.01 or ^∗∗∗^*P* < 0.001 indicated vs. Control group; ^##^*P* < 0.01 or ^###^*P* < 0.001 indicated vs. Model group.

Furthermore, to detect whether the Yinhuo Decoction affected the oxidative stress of the PC12 cells, we evaluated the indicators related to oxidative stress. In Figure 6, we found that the LDH, GPx, and SOD content of cells in the Model group were significantly increased, while ATP level was significantly decreased. However, Yinhuo Decoction reversed the expression of these indexes in CORT-treated PC12cells. The above evidence indicated that the Yinhuo Decoction inhibited the cellular oxidative stress.

### 3.7. Yinhuo Decoction Reduced CORT-Induced PC12 Damage by Regulating SIRT1/PGC-1*α* Pathway

**Figure 7:**
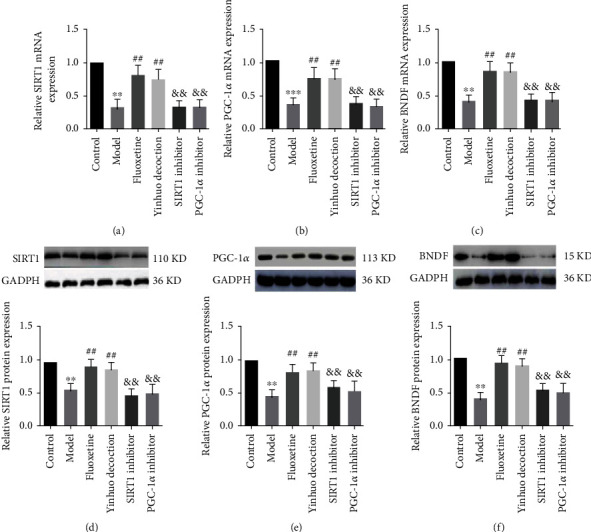
Yinhuo Decoction reduced CORT-induced PC12 damage by regulating SIRT1/PGC-1*α* pathway. (a) The mRNA expressions of SIRT1 in indicated groups were detected via qRT-PCR. (b) The mRNA expressions of PGC-1*α* in indicated groups were detected via qRT-PCR. (c) The mRNA expressions of BDNF in indicated groups were detected via qRT-PCR. (d) The protein expressions of SIRT1 were evaluated by western blot. (e) The protein expressions of PGC-1*α* were evaluated by western blot. (f) The protein expressions of BDNF were evaluated by western blot. SIRT1: silent mating type information regulation 2 hormolog 1; PGC-1*α*: peroxlsome proliferator-activated receptor-*γ* coactivator-1*α*; BDNF: brain-derived neurotrophic factor; ^∗∗^*P* < 0.01 or ^∗∗∗^*P* < 0.001 indicated vs. Control group; ^##^*P* < 0.01 or ^###^*P* < 0.001 indicated vs. Model group; ^&&^*P* < 0.01 indicated vs. Yinhuo Decoction group.

To investigate whether the protective mechanism of Yinhuo Decoction on PC12 cells was through the SIRT1/PGC-1*α* pathway, we detected the expression of related genes in this pathway. In addition, we also added pathway inhibitors for further confirmation. As shown in Figures 7(a)–7(c), the treatment of Yinhuo Decoction remarkably increased the mRNA expressions of SIRT1, PGC-1*α,* and BDNF as comparison to the Model group. Interestingly, when SIRT1 and PGC-1*α* inhibitors were added, the expression of SIRT1, PGC-1*α*, and BDNF mRNA in the PC12 cells was significantly reduced. Consistently, the western blot evidence confirmed that SIRT1 and PGC-1*α* inhibitors remarkably weakened the promoting effect of Yinhuo Decoction on SIRT1, PGC-1*α*, and BDNF protein expression (Figures 7(d)–7(f)).

## 4. Discussion

According to report, the HPA axis hyperfunction is one of the pathogenesis of depression, and most patients suffered with depression have different degrees of HPA axis dysfunction [12]. Besides, the HPA axis hyperactivity can elevate glucocorticoid and CORT levels, activate glucocorticoid receptors, and damage nerves in the brain, thus leading to depressive symptoms [21, 22]. It has been reported that the disturbance of neuroendocrine network induced by estrogen withdrawal in postmenopausal women can directly increase the incidence of depression [23, 24]. Here, we investigated the effect of Yinhuo Decoction on the HPA axis related indexes of PMD model mice and explore the inner link between Yinhuo Decoction and its correction of the HPA axis hypertrophy. In this study, our finding revealed that Yinhuo Decoction significantly increased adrenal coefficient and relieved adrenal lesions. Meanwhile, we observed that Yinhuo Decoction remarkably reduced the levels of CORT, total brain GCR, hippocampal GCR, and hypothalamus GCR. Collectively, the above evidence indicated that Yinhuo Decoction evidently improved the adrenal glands morphology and functions of PMD mice and reduced the CORT reactivity to correct the hyperactivity of HPA axis.

PC12 cells are derived from rat adrenal pheochromocytoma cells. Its morphology was similar to normal neurons, with conical shape and obvious axon, and some physiological functions are similar to normal neurons. In addition, available data proved that the survival rate of PC12 cells treated with high concentrations of CORT was lower than that of the normal group, and a similar effect was found in primary cultured hippocampal neurons treated with high concentrations of CORT [25, 26]. Thus, PC12 cells have been widely used as an in vitro model for both CORT-induced impairment of neuronal cells and their underlying molecular mechanisms. In this study, to explore the antidepressant effect of Yinhuo Decoction, we first screened the effective CORT treatment conditions and successfully replicated the cell mode. Previous studies have shown that exposure to light, drugs, or other stress can lead to decreased cell activity, DNA damage, and mitochondrial dysfunction, thus ultimately lead to nerve cell apoptosis [27]. In the study of Ohmoto et al., CORT produced significant growth suppression of retinoic acid-induced neurite outgrowth in N2A cells; however, Butein significantly increased neurite length and induced dose-dependent apoptotic cytotoxicity in N2A cells [28]. Consistently, our data revealed that CORT evidently inhibited the activity and DNA damaged of PC12 cells. More interesting, we observed that Yinhuo Decoction showed significant protective effect on CORT-damaged PC12 cells, representing as increased cell viability, and restored normal staining of cell nuclei. Taken together, Yinhuo Decoction effectively reduced the damage of CORT to cells by improving cell activity and reducing DNA injury.

One of the important signs of apoptosis is the decrease of mitochondrial membrane potential [29]. Sakthivel et al. proved that Phytol reduced A549 cells apoptosis by depolarizing the mitochondrial membrane potential [30]. Excitingly, our data also showed that the Yinhuo Decoction significantly increased the membrane potential in CORT-treated PC12 cell, which was consistent with previous study. Bcl-2 and Bax are the antiapoptotic and proapoptotic members of the Bcl-2 family, respectively. Bcl-2 prevents the apoptotic process through interaction with Bax to maintain mitochondrial membrane integrity [31], and caspase-3 acts as a critical effector in the apoptotic process [32]. In Zhang et al.'s study, they confirmed that Puerarin attenuated neurological deficits via Bcl-2/Bax/cleaved caspase-3 and Sirt3/SOD2 apoptotic pathways in subarachnoid hemorrhage mice [33]. Almutairi et al. demonstrated that SSNPs showed toxic effects on human liver cells via activating the caspase-3 activity, Bax activity, and inhibiting Bcl-2 activity [34]. Here, we proved that Yinhuo Decoction markedly inhibited CORT-induced apoptosis in PC12 cells by upregulating the expression of antiapoptotic protein Bcl-2 and downregulating the expression of proapoptotic protein Bax. Additionally, Yinhuo Decoction significantly inhibited the activation of caspase-3 in CORT-treated PC12 cells. Collectively, reducing apoptosis was another way that Yinhuo Decoction protected CORT-treated cells from injury.

Oxidative stress is believed to be associated with the advancement of depression, which was reported with abnormal changes in plenty of literatures [35, 36]. Besides, the high sensitivity of the brain to oxidative stress is mainly due to the relative lack of antioxidant enzymes, such as SOD and GPx, and high level LDH in tissues [37, 38]. For example, Roslan et al. showed that Quercetin ameliorated oxidative stress through inhibiting SOD, CAT, and GPx [39]. Wang et al. demonstrated that glycyrrhizin antagonized acute liver failure by inhibiting oxidative stress, presenting with downregulation of LDH [40]. Excitingly, our data revealed that Yinhuo Decoction evidently increased the content of SOD and GPx, while evidently reduced LDH in CORT-treated cells, suggesting that Yinhuo Decoction could effectively protect PC12 cells damaged by CORT through antioxidative stress. It was reported that the characteristics of the mechanism of depression are related to the existence of a low metabolic state (i.e., insufficient cellular energy) caused by damage to mitochondrial function [41]. Recent evidence has also linked the HPA axis to biological energy metabolism mechanisms, such as a decrease in ATP due to high levels of the HPA axis terminal hormone CORT in vivo [42]. Here, our data suggested that CORT reduced ATP level in PC12 cells, whereas Yinhuo Decoction effectively increased the level of ATP in CORT-damaged PC12 cells.

SIRT1 belongs to the class III histone deacetylase, which can deacetylate a variety of substrates and participate in a wide range of cellular processes, including energy metabolism, stress response, mitochondrial biogenesis and transformation, cell proliferation, differentiation, and survival [43, 44]. Currently, a large amount of research has shown that SIRTI can relieve depression-like symptoms. For example, Abe-Higuchi et al. depicted that after the addition of SIRT1 inhibitor, the level of SIRTI in the hippocampal DG of model mice decreased significantly, and depression-like behaviors appeared [2]. Lu et al. confirmed that SIRTI activator played an antidepressant effect [4]. Here, our data revealed that Yinhuo Decoction significantly increased the intracellular SIRTI level of CORT-treated cells. Conversely, when the SIRTI inhibitor was added, the intracellular SIRTI protein was evidently reduced, this was consistent with the previous results. According to report, SIRTI can activate transcription factors and metabolic coactivators, such as PGC-1*α*, by targeting selected genomic sites [45]. PGC-1*α* is a powerful transcriptional coregulator of mitochondrial genes, which can enhance the oxidative stress and mitochondrial respiration, and effectively improving the cognitive ability of rats [46]. Yang et al. found that exogenous IGF-1 alleviated depression-like behavior and hippocampal mitochondrial dysfunction via promoting the expression of PGC-1*α* [47]. Similarly, we also proved that Yinhuo Decoction increased the expression of PGC-1*α* in cells, and PGC-1*α* inhibitor reversed the expression. Brain-derived neurotrophic factor (BDNF), as a highly expressed neurotrophic factor, is involved in many aspects of brain development. A reduction of BDNF can increase anxiety and depression-like symptoms, which were also associated with depression severity and recurrence [48]. Shen et al. proved that Berberine improved the depression-like behavior of mice induced by CORT by upregulating hippocampal BDNF expression [49]. Consistent with previous study, our results also confirmed that Yinhuo Decoction increased the protein expression of BDNF in CORT-treated cells. Taken together, Yinhuo Decoction can improve depression by regulating SIRTI/PGC-1*α* signaling pathway.

## 5. Conclusion

**Figure 8:**
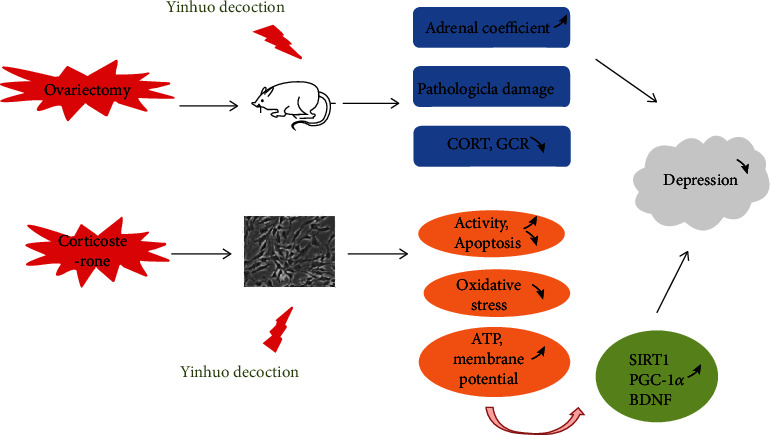
The diagram of the mechanism of Yinhuo Decoction acting on depression in vivo and in vitro.

In this study, we concluded that Yinhuo Decoction improved the adrenal glands morphology and functions of PMD mice and reduced the CORT reactivity to correct the hyperactivity of HPA axis. Besides, Yinhuo Decoction antagonized CORT-induced injury in PC12 cells via activating the SIRT1/PGC-1*α* signaling pathway (Figure 8).

## Data Availability

The data used to support the findings of this study are available from the corresponding author upon request.
